# Frequency dependent whole-brain coactivation patterns analysis in Alzheimer’s disease

**DOI:** 10.3389/fnins.2023.1198839

**Published:** 2023-10-25

**Authors:** Si-Ping Zhang, Bi Mao, Tianlin Zhou, Chun-Wang Su, Chenxi Li, Junjie Jiang, Simeng An, Nan Yao, Youjun Li, Zi-Gang Huang, Alzheimer’s Disease Neuroimaging Initiative

**Affiliations:** ^1^The Key Laboratory of Biomedical Information Engineering of Ministry of Education, Institute of Health and Rehabilitation Science, School of Life Science and Technology, Xi'an Jiaotong University, The Key Laboratory of Neuro-Informatics & Rehabilitation Engineering of Ministry of Civil Affairs, Xi'an, Shaanxi, China; ^2^Research Center for Brain-Inspired Intelligence, Xi'an Jiaotong University, Xi'an, Shaanxi, China; ^3^Department of Military Medical Psychology, Air Force Medical University, Xi’an, Shaanxi, China; ^4^Department of Applied Physics, Xi'an University of Technology, Xi'an, China; ^5^The State Key Laboratory of Cognitive Neuroscience and Learning, Beijing Normal University, Beijing, China

**Keywords:** coactivation pattern, subbands, resting state fMRI, Alzheimer’s disease, MMSE score

## Abstract

**Background:**

The brain in resting state has complex dynamic properties and shows frequency dependent characteristics. The frequency-dependent whole-brain dynamic changes of resting state across the scans have been ignored in Alzheimer’s disease (AD).

**Objective:**

Coactivation pattern (CAP) analysis can identify different brain states. This paper aimed to investigate the dynamic characteristics of frequency dependent whole-brain CAPs in AD.

**Methods:**

We utilized a multiband CAP approach to model the state space and study brain dynamics in both AD and NC. The correlation between the dynamic characteristics and the subjects’ clinical index was further analyzed.

**Results:**

The results showed similar CAP patterns at different frequency bands, but the occurrence of patterns was different. In addition, CAPs associated with the default mode network (DMN) and the ventral/dorsal visual network (dorsal/ventral VN) were altered significantly between the AD and NC groups. This study also found the correlation between the altered dynamic characteristics of frequency dependent CAPs and the patients’ clinical Mini-Mental State Examination assessment scale scores.

**Conclusion:**

This study revealed that while similar CAP spatial patterns appear in different frequency bands, their dynamic characteristics in subbands vary. In addition, delineating subbands was more helpful in distinguishing AD from NC in terms of CAP.

## 1. Introduction

Alzheimer’s disease (AD) is a typical neurodegenerative disease among older people, and its incidence is increasing every year. In the early stages of AD, individuals experience a decline in memory function. This decline is accompanied by progressive damage to various higher cognitive functions, ultimately leading to a gradual loss of the ability to live independently and, eventually, death. The prevailing conventional view is that AD is mainly associated with excessive deposition of extracellular beta-amyloid protein (Aβ) in neurons and hyperphosphorylation of Tau protein in neuronal cells. The excessive accumulation of Aβ protein in the brain forms extracellular plaques or senile plaques, which have toxic effects on neurons. They can have a complex impact on a patient’s brain function (Masters et al., 2015). Therefore, the identification of brain abnormalities specific to the early stages of AD is crucial to the study of brain injury in AD.

Functional magnetic resonance imaging (fMRI) provides spatial and temporal information about the spontaneous activity of the brain, and its high spatial resolution allows us to explore the interactions between brain regions. Resting-state functional MRI (rs-fMRI) is the measurement of low-frequency fluctuations (LFFs) in the brain’s blood oxygen level-dependent (BOLD) signal. This helps reflect the brain’s spontaneous neural activity in the absence of a specific task (Raichle et al., 2001; Grodd and Beckmann, 2014). It has become one of the main imaging tools for exploring the neural mechanisms of the brain. Resting-state functional MRI-based brain function analysis is frequently employed in clinical research for various neurological disorders, including Alzheimer’s disease (Barkhof et al., 2014), Parkinson’s disease, schizophrenia, depression, and more (Griffanti et al., 2016; Khazaee et al., 2016, 2017; Wang et al., 2016). Especially for some patients with cognitive impairment, it is difficult to perform cognitive tasks during MRI scanning. Therefore, resting-state fMRI provides an effective way to study the brain function of AD patients (Dosenbach et al., 2010).

Current methods of analyzing resting-state fMRI data, such as functional connectivity (FC), are often based on the assumption that brain activity is at a steady state (Arbabshirani et al., 2013). In recent years, it has been found that resting brain activation is not stable; instead, it transitions among multiple patterns. Moreover, the corresponding activation patterns and transition behaviors of patients with brain dysfunction were significantly different from those of healthy subjects (Griffanti et al., 2016; Khazaee et al., 2016, 2017; Wang et al., 2016). Therefore, considering only static functional connectivity is not sufficient to explain the time-varying dynamic information interactions of the brain; rather, the dynamics of functional connectivity should be investigated to reveal the complex and variable mechanisms of brain networks. In recent years, a number of dynamic functional connectivity (dFC) methods have been developed that focus on the time-varying properties of functional connectivity in the brain. Sliding window correlation (SWC) is the most widely used method (Chang and Glover, 2010; Allen et al., 2014). It reflects the dynamics of brain networks by calculating functional connectivity over different periods of time. However, the sliding window method suffers from many limitations (Hindriks et al., 2016). In particular, the choice of window length is crucial. Long windows are insensitive to rapid changes in connectivity, while short windows are more susceptible to high-frequency noise fluctuations (Leonardi and Van De Ville, 2015). Therefore, many alternatives to the sliding window method have emerged, such as time-frequency domain-based analysis, point process analysis (PPA) methods (Liu and Duyn, 2013; Chen et al., 2015), sliding-window methods based on temporal functional modes (TFMs; Smith et al., 2012), PPA-inspired coactivation patterns (CAPs; Liu and Duyn, 2013; Liu et al., 2018), etc.

In contrast to the sliding window-based approach, which considers spontaneous brain activity to be slowly changing, Tagliazucchi et al. proposed that information related to resting brain activity can be tied to discrete events and that point process analysis (PPA) which includes only relevant time points can contain the same information as conventional full-time course analysis (Tagliazucchi et al., 2011, 2012, 2016). Based on this idea that meaningful information can actually be obtained from the observation of individual frames, a new alternative to functional connectivity analysis is proposed.

Inspired by the PPA method, Liu and Duyn (2013) and Liu et al. (2018) extracted time frames with activation levels above a specific threshold in the region of interest (ROI) and obtained CAPs by a clustering algorithm. These CAPs are highly similar to those obtained by correlation analysis. Subsequent improvements to the CAP technique have been developed, such as the extension to a data-driven whole brain analysis method (Liu et al., 2013). This allows for the generation of CAPs with different activation patterns and avoids the effects of selecting a specific activation threshold of the ROI.

In addition, the brain has complex dynamic properties and is capable of generating oscillatory waves at many different frequencies. Synchronous oscillatory activities of neural networks are regarded as the key to realizing the function. Frequency-specific studies initiated by Buzsaki et al. found that neural networks in the mammalian forebrain display several oscillatory bands, and the average frequency of oscillations forms a linear relationship on the natural logarithmic scale. The adjacent frequencies were separated with a constant ratio. They suggested that the separate bands are generated by different oscillators, each possessing specific properties and physiological functions (Penttonen and Buzsáki, 2003; Buzsáki and Draguhn, 2004). This extended Buzsaki’s framework to low-frequency oscillations of the BOLD signal, subdividing the spontaneous BOLD waves into four different frequency ranges. Slow-2 (0.198–0.25 Hz) and slow-3 (0.073–0.198 Hz) were mainly confined to the white matter. Slow-4 (0.027–0.073 Hz) and slow-5 (0.01–0.027 Hz) oscillations were clearly detected in the gray matter. The respiratory and heart signals were in the range of slow 2–3, while the oscillations underlying the resting-state functional connectivity were mainly in the range of slow 4–5 (Zuo et al., 2010). Several studies have shown that the low-frequency oscillation pattern and functional connectivity in MCI and AD is frequency dependent (Han et al., 2011; Wang et al., 2011; Liu et al., 2014; Li et al., 2017). The pattern of disruption of spontaneous neural activity and the ALFF/fALFF of brain regions may also differ between subbands (slow-4 and slow-5; Han et al., 2011; Liu et al., 2014). It has also been proposed that classifiers trained by fusing multiband ALFF and fALFF features have good performance in distinguishing NC, MCI and AD (Yang et al., 2018). In addition, it has been found that band segmentation significantly improves the accuracy of MCI classifiers using graph theory-based functionally connected networks (Wee et al., 2012). Another study found that the coupling between global functional connectivity (FC) and low frequency oscillatory amplitude (ALFF) in the brains of dementia patients showed abnormalities/losses in certain brain regions compared to healthy subjects and that changes in this coupling were sensitive to band range (Mascali et al., 2015). Therefore, it seems necessary to further investigate the specific changes in brain regions in different frequency bands.

In this study, we used a multiband CAP approach to model the state space and study brain dynamics in AD and NC. Unlike previous studies, we focus on the frequency dependence of the CAP. fMRI data in the original frequency band of 0.01–0.1 Hz (LFO band) were subdivided into the slow-5 band (0.01–0.027 Hz) and slow-4 band (0.027–0.073 Hz) to calculate the CAP. In addition, we used classical dynamic features of state occupancy (Occurrence), mean duration (Duration) and frequency of occurrence (EntryRate) of CAPs to measure changes in the dynamics of resting-state brain spontaneous activity. We further analyzed the correlation between the dynamic characteristics and the subjects’ clinical indices to explore potential relationships between altered brain dynamics and disease pathology.

## 2. Materials and methods

### 2.1. Participants

The subject fMRI data for this study were obtained from the Alzheimer’s Disease Neuroimaging Initiative (ADNI) database. ADNI is an open database resource available to all researchers around the world.^1^ The downloaded data for the subjects included 3 T structural and functional MRI data and clinical index. The subjects were diagnosed with AD or normal control by the Mini-Mental State Examination (MMSE) and Clinical Dementia Rating Scale (CDR) scores. In this study, we selected 42 AD patients and 42 healthy elderly individuals from the database, excluding 12 subjects based on head motion parameters and registration results. The detailed demographic information is shown in Table 1.

**Table 1:** Demographic and clinical information of subjects.

	AD (*n* = 33)	NC (*n* = 39)	*p* value
Sex(female/male)	14/19	16/23	0.90
Age(mean ± SE)	73.35 ± 1.30	74.44 ± 1.07	0.51
MMSE(mean ± SE)	22.97 ± 0.42	28.87 ± 0.20	< 0.001
CDR(mean ± SE)	0.76 ± 0.08	0.00 ± 0.00	< 0.001

Participants in this study were selected from the ADNI2. Resting-state functional MRI and T1 data were all obtained with a 3.0 T Philips machine for consistency. The acquisition parameters were as follows: fMRI Scan. The scan parameters for the EPI fast imaging sequence were as follows: flip angle = 80°, matrix = 64 × 64 × 6,720, slice thickness = 3.3 mm, TR = 3,000 ms, TE = 30 ms, pixel spacing = 3.3 × 3.3 × 3.3 mm3. The scan parameters for the 3D weighted T1 structure image were as follows: acquisition plan = sagittal, flip angle = 9°, matrix = 256 × 256 × 170, slice thickness = 1.2 mm, TR = 6.67 ms, TE = 3.1 ms, pixel spacing = 1 × 1 × 1.2 mm^3^.

### 2.2. Data preprocessing

Resting-state functional MRI data were preprocessed by the FSL toolkit and the AFNI toolkit. The preprocessing process was as follows: (1) removal of the first four time points to ensure that the data were from a stable magnetic field, (2) application of the 3dvolreg program of AFNI for each subject for head-motion correction (transition >2 mm or rotation >2°, 12 subjects were excluded), and spatially smoothed data by a Gaussian-distributed smoothing kernel with a half-peak width of 6 mm, (3) slice timing correction, (4) detrending linear trends and bandpass filtering to three frequency bands (0.01–0.027 Hz, 0.027–0.073 Hz, 0.01–0.1 Hz), (5) alignment of each subject’s preprocessed fMRI to the MNI152 standard spatial template using the linear alignment FLIRT algorithm, and (6) regression of white matter signal and cerebrospinal fluid signal.

### 2.3. Whole brain coactivation pattern analysis

**Figure 1:**
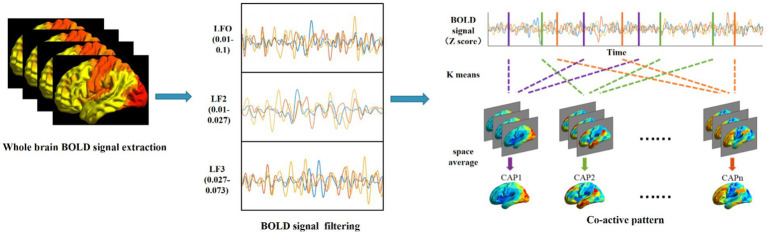
Schematic diagram of the coactivation mode (CAP) calculation process.

For all participants, we applied k-means clustering algorithms to identify whole-brain CAP with voxel resolution in three different frequency bands. The computational process for CAP is shown in Figure 1. The preprocessed fMRI data from three frequency bands are clustered separately. This division results in k clusters, and the spatial patterns corresponding to all fMRI frames within each cluster are averaged to generate a CAP. The four-dimensional fMRI data of each subject are formatted into T*N-dimensional vectors, where T is the number of frames and N is the number of voxels, and a single N-dimensional vector is the basic unit of k-means clustering. To determine the stable cluster result, we first subsampled the frames of each subject along the time dimension by calculating the variance of the N-dimensional vector within each frame. According to the method suggested by Allen et al. (2014), subject exemplars were chosen as the frames with local maxima in variance. The number of exemplar frames for each subject ranged from 10 to 15. In our study, the k-means algorithm was iterated 500 times on the subsampling frames, and the resulting centroids were then utilized as the initial value for clustering all time frames of subjects. The silhouette method was used to evaluate the clustering performance with numbers ranging from 2 to 20, and the optimal number of clusters was determined to be seven based on maximal silhouette across all the iterations, which was consistent with our previous research (Li et al., 2022).

### 2.4. Time-varying characterization of dynamic brain network states

The different CAPs obtained by clustering the data in different frequency bands represent different states of spontaneous brain activity in the resting state. The activity pattern of each state is described by calculating occurrence, entry rate, mean duration and transition probability in different frequency bands. This reflects the resting brain functional network state characteristics of AD patients. Occurrence indicates the proportion of each CAP across all scans; entry rate refers to the number of times a state occurs, with consecutive occurrences counted as 1. Mean duration indicates the average length of CAP that can be maintained before it transitions to the next. Transition probability indicates how often a state transitions from one to another. More transitions reflect that the system is unstable. The number of CAPs is determined by the proportion of NC, with CAPs ranked from 1 to 7 in decreasing order of proportion.

### 2.5. Statistical analysis

Subject demographic and clinical index information was statistically analyzed using SPSS26, with chi-square tests for sex and one-way analysis of variance (ANOVA) statistical methods for age. For the characteristics of CAPs (occurrence, entry rate, mean duration and transition probability, etc.), the independent samples *t*-test was used to analyze the statistical significance of the differences between groups, with statistical significance set at *p* < 0.05. Pearson correlations were performed between the characteristics of CAPs and the clinical MMSE index (*p* < 0.05).

## 3. Results

**Figure 2:**
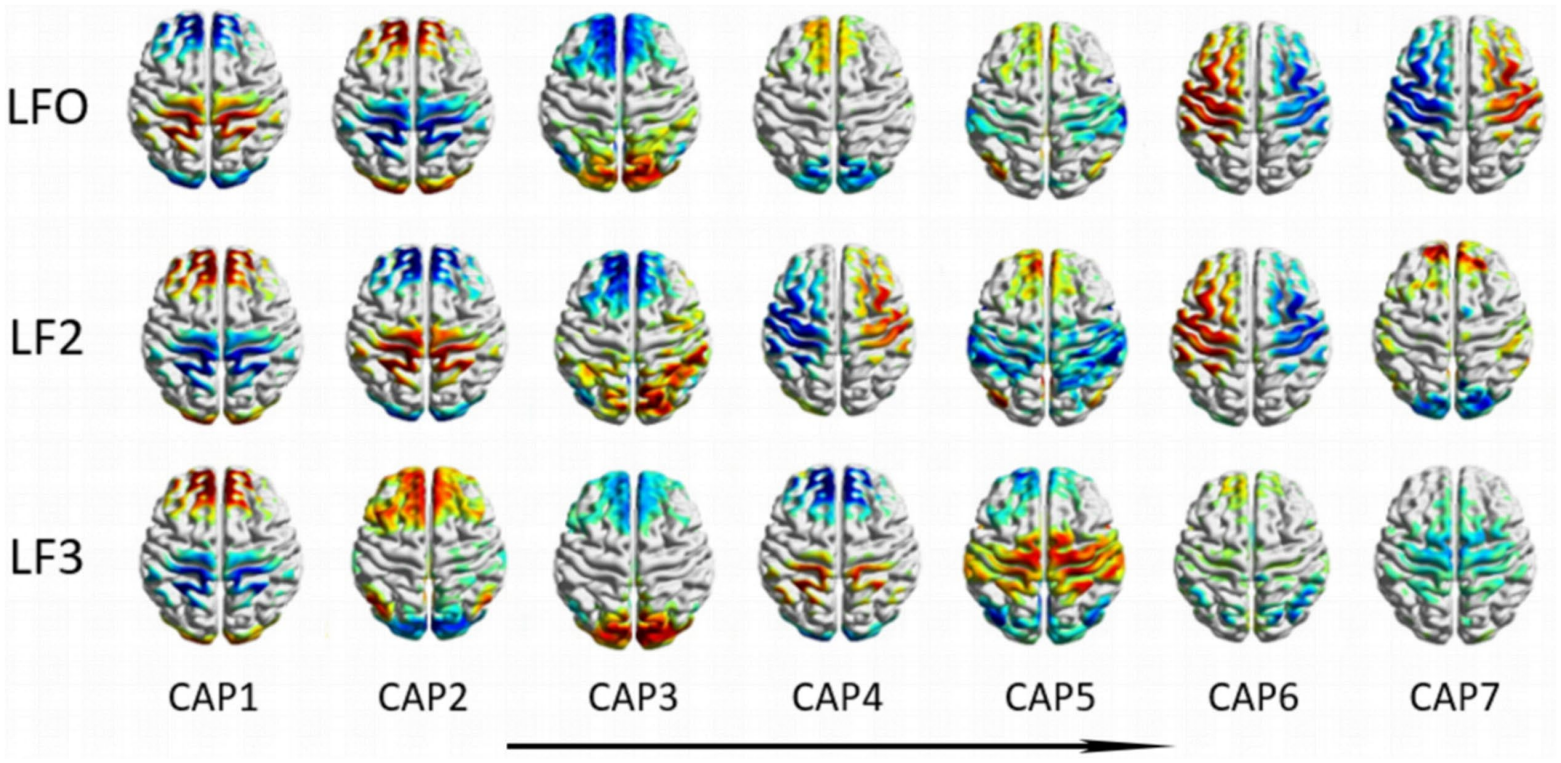
Coactivation mode profiles at different frequency bands. The seven CAP patterns in different frequency bands are shown vertically from left to right, where the order is determined by the proportion of occurrence of CAPs among NC subjects in descending order.

A total of 21 CAPs in three different frequency bands are shown in Figure 2. The seven CAP patterns in different frequency bands are shown vertically from left to right, where the order is determined by the proportion of occurrence of CAPs among NC subjects in descending order, i.e., CAP 1 has the highest proportion, and CAP 7 has the lowest proportion. We calculated the Z score for each CAP, and the value of voxels above positive or negative 1.5 times the standard deviation are shown in Figure 2.

### 3.1. LFO band

**Figure 3:**
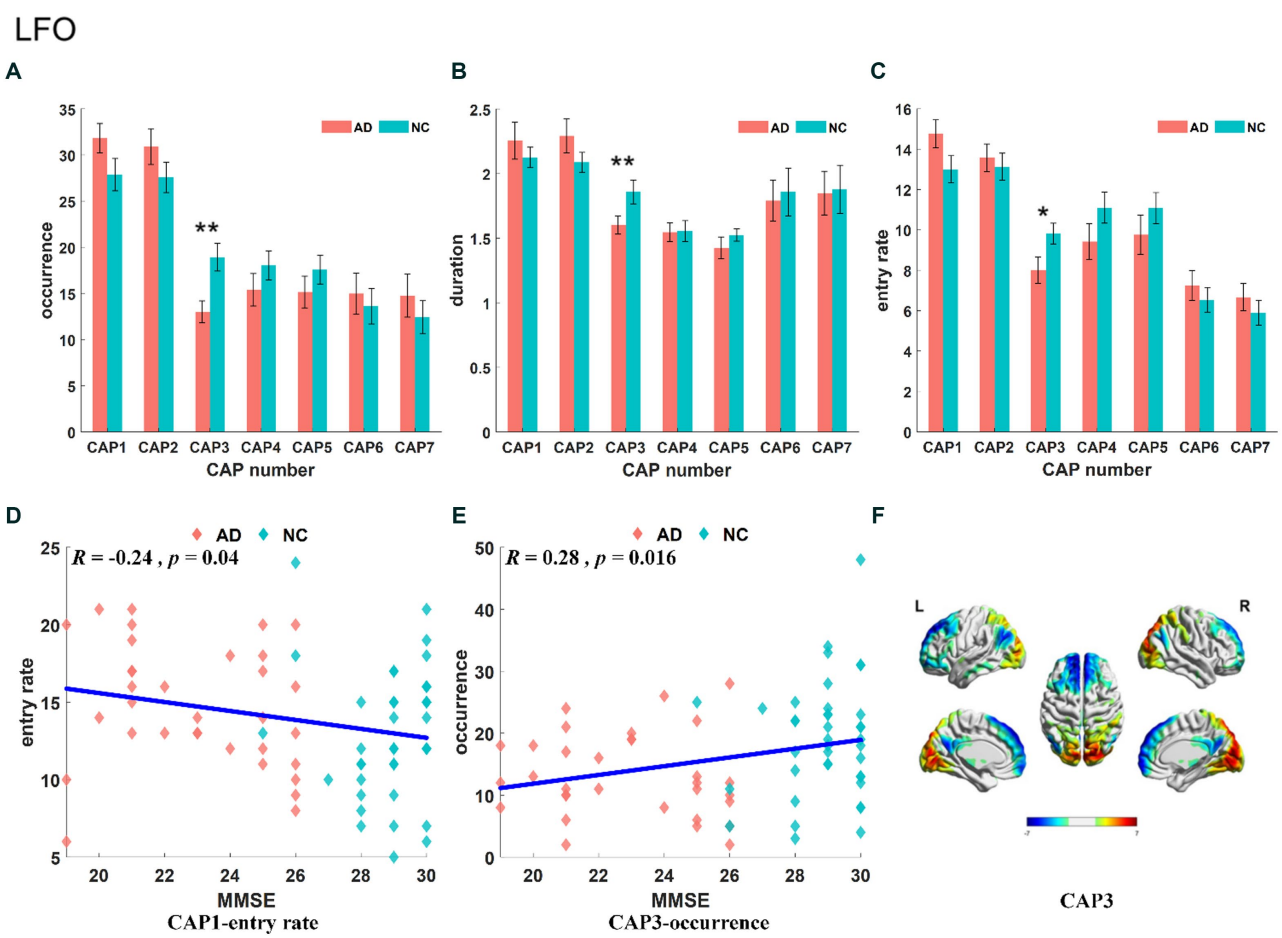
Statistical results of time-varying characteristic in the LFO band. The comparison of occurrence between AD and NC in different CAPs **(A)**. Duration of AD vs. NC in different CAPs **(B)**. Entry rate of AD vs. NC in different CAPs **(C)**. The correlation between entry rate for CAP1 and MMSE **(D)**. The correlation between occurrence for CAP3 and MMSE **(E)**. The spatial mapping of CAP3 in the LFO band **(F)**.

The results of the statistical analysis of the time-varying characteristics of each CAP in the LFO band are shown in Figures 3A–C. We only found that the characteristics of CAP3 were significantly decreased in the AD group compared with the NC group, including occurrence (*p* < 0.01), duration (*p* < 0.01) and entry rate (*p* < 0.05). The frequency of CAP1 was negatively correlated with subjects’ MMSE score (Figure 3D), and the occurrence of CAP3 was positively correlated with subjects’ MMSE scores (Figure 3E). The precuneus and superior parietal lobule showed activation in CAP1, mainly in areas 5 and 7 of Brodmann’s area, while inhibitory brain areas were mainly in the superior frontal gyrus, medial frontal gyrus, calcarine gyrus, lingual gyrus, occipital gyrus and cuneus, located in areas 9/10/17/18 of Brodmann’s area.CAP2 has opposite activation patterns to CAP1. The spatial mapping of CAP3 mainly showed activation and inhibition in the primary visual cortex, default mode network and prefrontal-related brain regions (Figure 3F). Activated brain areas include the talar sulcus, lingual gyrus, occipital gyrus, cuneus and precuneus, primarily in regions 17 and 19 lingual of the Brodmann areas, while inhibitory brain areas are mainly in the prefrontal lobe, including the superior frontal gyrus, medial frontal gyrus and middle frontal gyrus, concentrated in regions 9 and 10 of the Brodmann areas. CAP6-CAP7 also exhibited opposite activation patterns. The activated brain region of CAP6 corresponded to the inhibitory brain region of CAP7, primarily involving the left middle frontal gyrus, right occipital gyrus, and right temporal gyrus, and located in areas 8/18/19/20 of Brodmann’s area. Conversely, the inhibitory brain region of CAP6 corresponded to the activated brain region of CAP7, mainly comprising the right middle frontal gyrus, left occipital gyrus and left temporal gyrus, these brain regions exhibited symmetry to the activated brain region.

### 3.2. Slow-5 band

**Figure 4:**
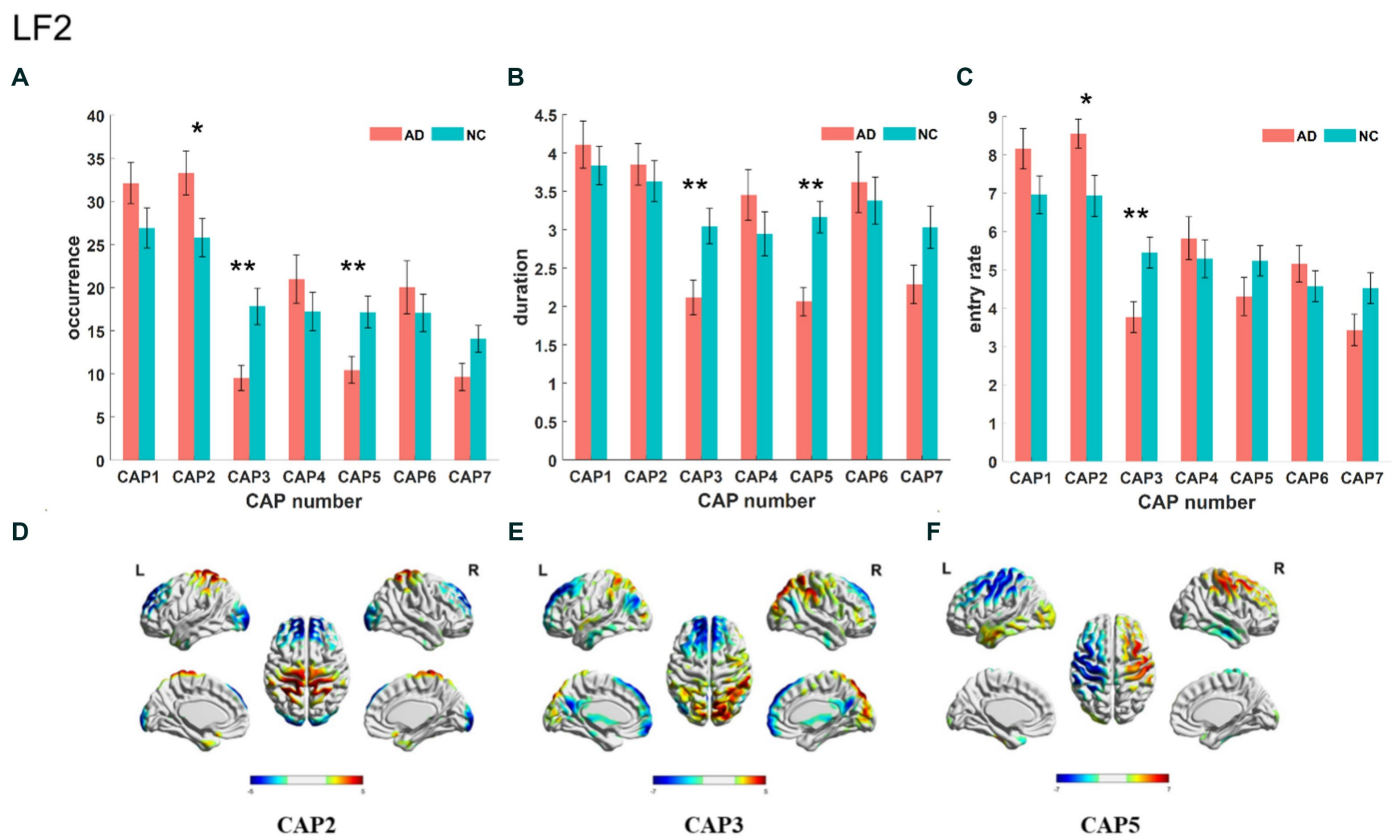
Statistical results of time-varying characteristic in the slow-5 band. The comparison of occurrence between AD and NC in different CAPs **(A)**. Duration of AD vs. NC in different CAPs **(B)**. Entry rate of AD vs. NC in different CAPs **(C)**. The spatial mapping of CAP2/3/5 in the slow-5 band **(D–F)**.

**Figure 5:**
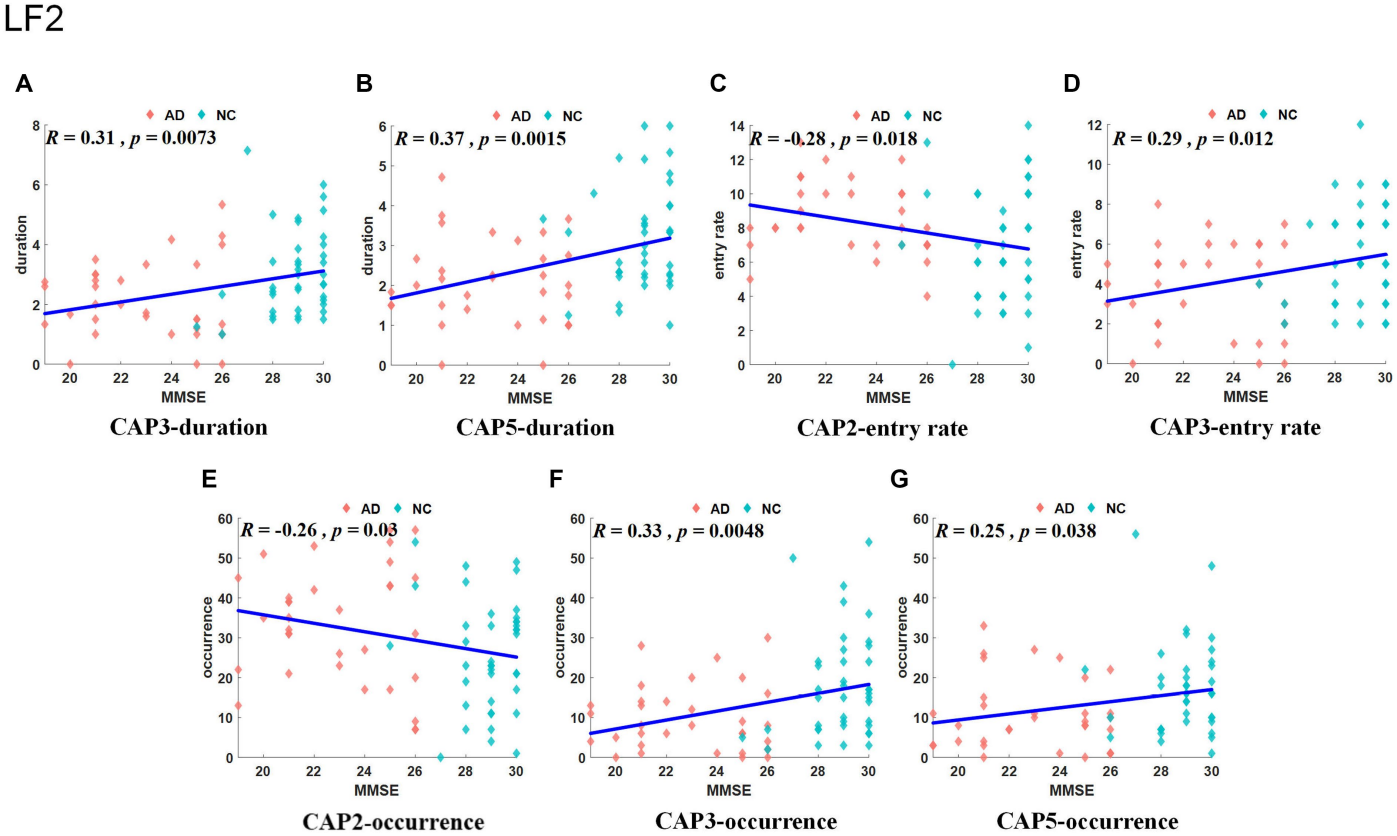
Results of correlation analysis between typical CAP and MMSE scores in the slow-5 band. The correlation between duration for CAP3/5 and MMSE **(A, B)**. The correlation between entry rate for CAP2/3 and MMSE **(C, D)**. The correlation between occurrence for CAP2/3/5 and MMSE **(E–G)**.

The Slow-5 band is the low-frequency part of the full band. As shown in Figure 4, the occurrence of CAP3 and CAP5 was significantly decreased in the AD group, but the occurrence of CAP2 was increased. Regarding characteristic duration, both CAP3 and CAP5 significantly decreased in the AD group when compared with the NC group. The entry rate of CAP3 was decreased, and CAP2 was increased in the AD group. CAP1-CAP2 have opposite activation patterns. The precuneus and superior parietal lobule show inhibition in CAP1 and activation in CAP2. These regions belong to the default mode network, primarily in regions 5 and 7 of the Brodmann areas. In contrast, the activated brain areas of CAP1 correspond to the inhibited brain areas of CAP2, mainly including the superior frontal gyrus, medial frontal gyrus, calcarine gyrus, lingual gyrus and occipital gyrus, concentrated in parts of the prefrontal lobule and primary visual cortex, regions 9/10/17, among others, of the Brodmann areas. CAP3 in this band is similar to CAP2, although CAP3 is also inhibitory in the temporal gyrus and angular gyrus of the temporal cortex (Brodmann area, BA 39) and in the posterior cingulate cortex (BA 23). The activated brain regions of CAP4 mainly contain the right inferior parietal lobule (BA 40), right postcentral gyrus (BA 2), right middle frontal gyrus (BA 9), left occipital gyrus, and left temporal gyrus, while the inhibitory brain regions are symmetrical to the activated brain regions. CAP5 mainly shows inhibition in the precentral gyrus and postcentral gyrus (BA 3 and 4) of the left brain but activation in the right, mainly containing the posterior cingulate (BA 23), the precuneus (BA 23), the angular gyrus, the occipital gyrus, and the middle orbital gyrus. The results of the correlation analysis of the CAP characteristics with the subjects’ MMSE scores are shown in Figure 5, which indicates that the altered characteristics of CAPs almost showed significant correlations with the clinical index. All characteristics of the CAP3 and the occurrence and duration of the CAP5 showed significant positive correlations with the MMSE. In contrast, the occurrence and entry rate of CAP2 showed significant negative correlations with MMSE scores.

### 3.3. Slow-4 band

**Figure 6:**
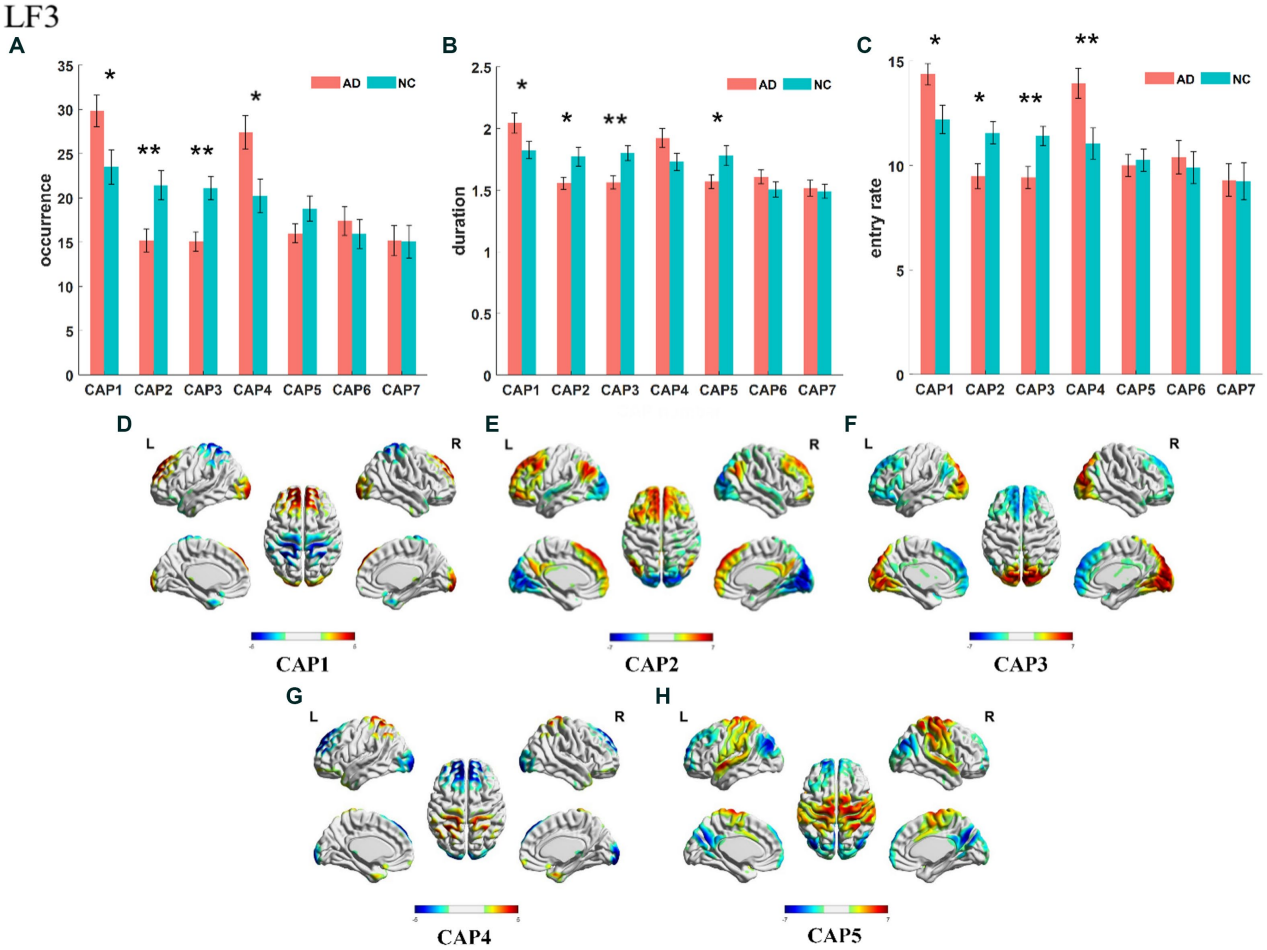
Statistical results of time-varying characteristic in the slow-4 band. The comparison of occurrence between AD and NC in different CAPs **(A)**. Duration of AD vs. NC in different CAPs **(B)**. Entry rate of AD vs. NC in different CAPs **(C)**. The spatial mapping of CAP1/2/3/4/5 in the slow-4 band **(D–H)**.

**Figure 7:**
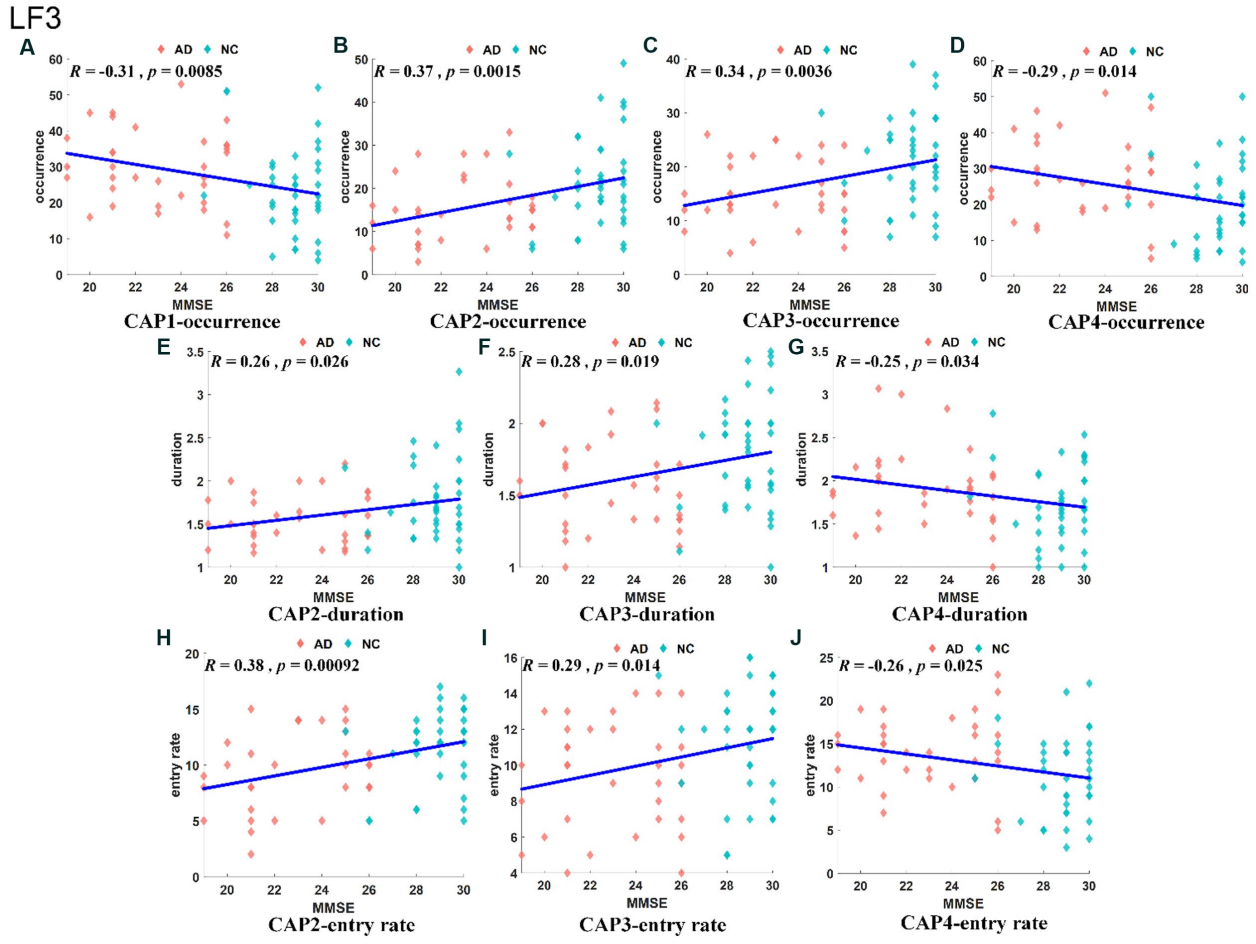
Results of correlation analysis of typical CAP and MMSE scores in the slow-4 band. The correlation between occurrence for CAP1/2/3/4 and MMSE **(A–D)**. The correlation between duration for CAP2/3/4 and MMSE **(E–G)**. The correlation between entry rate for CAP2/3/4 and MMSE **(H–J)**.

The slow-4 band (0.027–0.073 Hz) is the high-frequency part of the full band. The occurrence, duration and entry rate of CAP1 in the slow-4 band were increased in the AD group compared with the NC group; however, those of CAP2 and CAP3 were decreased. In addition, the occurrence and entry rate of CAP4 were increased, and the duration of CAP5 was decreased (Figure 6). The activation patterns of CAP1-CAP4 in the slow-4 band are opposite. In CAP1, the main activation regions are the superior media and frontal gyrus (BA 9 and 10), while the inhibition regions are the precuneus and superior parietal lobe (BA 5 and 7), and CAP4 is the opposite. In CAP2, the main activated regions include the superior frontal gyrus, medial frontal gyrus, angular gyrus, anterior cingulate cortex, and posterior cingulate, concentrated in Brodmann’s area in areas 9/23/39, etc., while the inhibition regions are the cuneus (BA 17) and lingual gyrus (BA 18). The activation pattern of CAP3 is mainly opposite to CAP2, with activation in the posterior cingulate (BA 23), cuneus (BA 17) and lingual gyrus (BA 18) and inhibition in the superior frontal gyrus (BA 9), medial frontal gyrus (BA 9), angular gyrus (BA 39) and anterior cingulate cortex. In CAP5, the main activation region is the postcentral/precentral gyrus (BA 3 and 4), and the inhibition region includes the superior medial/frontal gyrus (BA 9 and 10), angular/occipital gyrus (BA 39) and precuneus/posterior cingulate (BA 7), as shown in Figure 7. Most of the altered characteristics in the slow-4 bands that significantly changed exhibited significant correlations with the cognitive index except for the duration and entry rate of CAP1 as shown in Figure 7.

### 3.4. Cross-band CAP correlations

**Figure 8:**
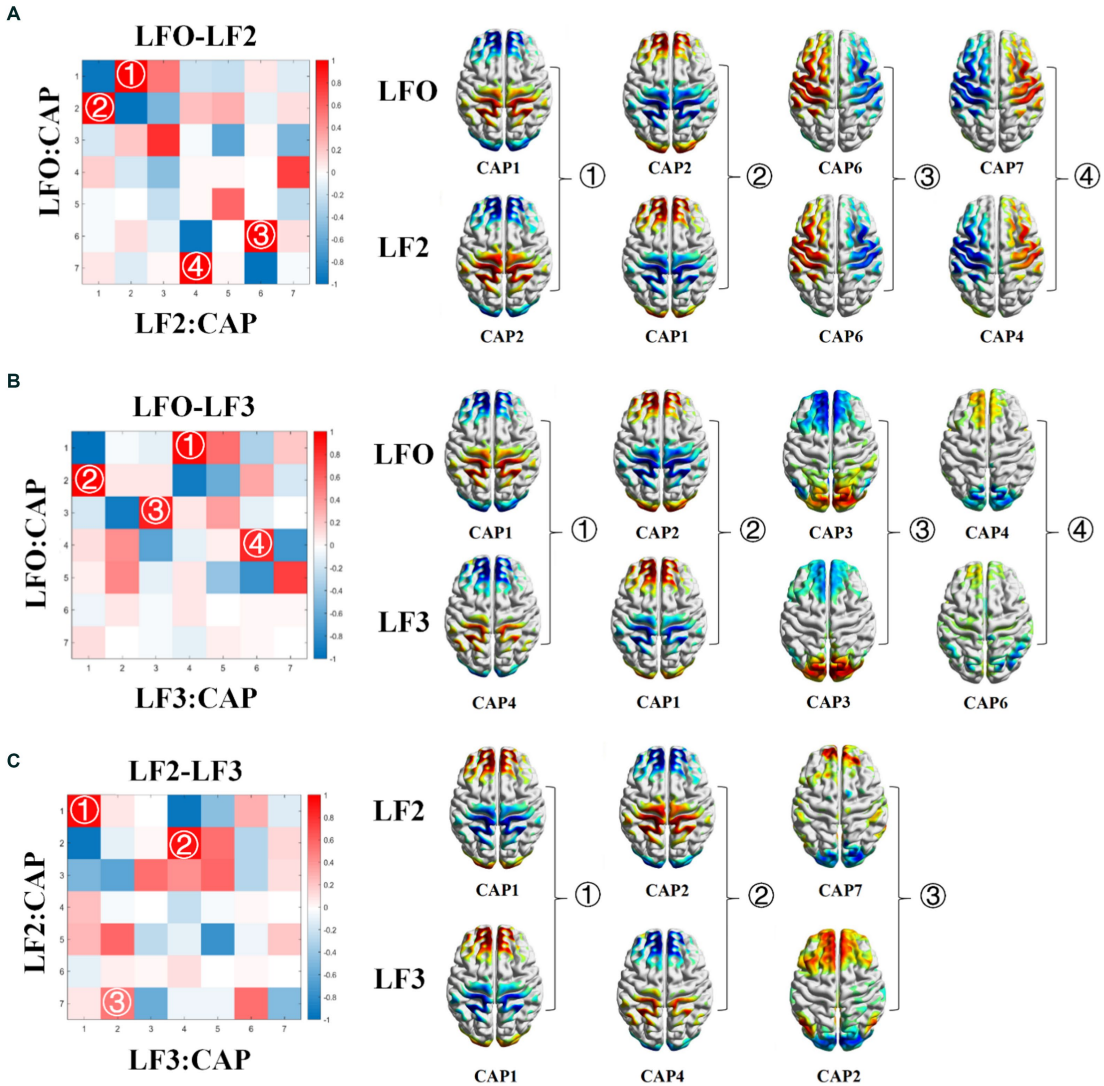
CAP cross-band correlation matrix. The correlation matrix between LFO band and slow-5 band **(A)**. The correlation matrix between LFO band and slow-4 band **(B)**. The correlation matrix between slow-5 band and slow-4 band **(C)**.

From Figure 2, the CAPs in different frequency bands show similar patterns. To further explore the cross-band correlation of CAP activation patterns, we calculated the Pearson correlation of different CAPs between the two bands separately and obtained three CAP cross-band correlation matrices, as shown in Figure 8. From the matrix, we can find many elements with large correlation values or even close to one, such as CAP2 in the LFO band and CAP1 in the slow-5 band, CAP7 in the LFO band and CAP4 in the slow-5 band. These results indicate that the CAPs between different bands have similar patterns. However, it is intriguing to note that highly similar CAPs in different bands do not have the same number, which indicates that the similar CAPs between different bands do not maintain the same order of occurrence.

## 4. Discussion

Our study focused on resting-state fMRI data of patients with AD using whole-brain CAP analysis in multiple frequency bands. The findings revealed similar CAP patterns at different frequency bands, but the occurrence of patterns was different. Furthermore, there were significant alterations in CAPs associated with the default mode network (DMN) and the ventral/dorsal visual network (dorsal/ventral VN) when comparing the AD and NC groups. The findings also revealed that delineating subbands was more helpful in distinguishing AD from NC in terms of CAP.

The spatial mapping of all CAPs at different frequency bands and the results of the cross-band correlation matrix indicated that many CAPs between different frequency bands were highly similar. However, the occurrence of similar CAPs between different frequency bands was not consistent. For example, CAP 1 in the LFO band, CAP 2 in the slow-5 band, CAP 7 in the LFO band and CAP 4 in the slow-5 band exhibit highly similar spatial patterns, but their occurrence patterns differ. Conversely, CAPs like CAP 3 in the LFO band and CAP 6 in the slow-5 band share similar patterns and the same occurrence order. Similar results are seen between the LFO-slow-4 and slow-5-slow-4 bands. The resting brain constantly switches between multiple steady states (Chang and Glover, 2010; Smith et al., 2012; Liu et al., 2013), and CAP divide spontaneous activity in the resting brain into multiple substates, i.e., patterns of repeated coactivation or deactivation of different brain regions, based on differences in the transient spatial activation patterns of the brain. Previous studies have shown that there are both similarities and differences in the activation of brain regions and network connections of different frequency bands in AD patients. Our results were consistent with these results. Each CAP represents a state of spontaneous brain activity in the resting state of the brain, and a similar pattern of brain activity can be repeated in different frequency bands, but the occurrence was altered. At present, there are relatively few studies on the use of the frequency dependent whole-brain CAP method in the analysis of resting-state fMRI data for neurological diseases. Hang et al. used the Frequency dependent CAP method to study schizophrenia and found that the spatial patterns of CAP remained consistent across different frequency bands, which is familiar with our findings (Yang et al., 2022).

The characteristics of CAPs associated with the DMN and ventral/dorsal visual network were altered significantly between the AD and NC groups. Some CAP patterns with activation or inhibition of DMN network-related brain areas were significantly different between AD and NC subjects. The CAP3 spatial map in the LFO band showed activation and inhibition in the primary visual cortex, the default mode network and prefrontal-related brain regions. The activated brain areas include the talar sulcus, lingual gyrus, occipital gyrus, cuneus and precuneus, which are mainly associated with some higher brain functions, such as episodic memory, visuospatial processing, self-reflection and consciousness (Cavanna and Trimble, 2006; Xia et al., 2013). In CAP3, inhibitory brain areas encompass portions of the prefrontal lobe, including the superior frontal gyrus, medial frontal gyrus, and middle frontal gyrus, primarily concentrated in Brodmann areas 9 and 10. These regions are associated with higher-order cognitive functions like working memory and self-awareness (Boisgueheneuc et al., 2006). In addition, there was a significant positive correlation between the state percentage of CAP3 and the cognitive level of the subjects. The patterns that were altered in the slow-5 band were CAP2, CAP3 and CAP5. Although, CAP3 also showed inhibition in the temporal gyrus and angular gyrus of the temporal cortex (area 39 of Brodmann’s subdivision) and in the posterior cingulate cortex (area 23 of Brodmann’s subdivision). The posterior cingulate cortex is the central node of the default mode network, which is connected to intrinsic control networks and more active during periods of inattention (e.g., recall of episodic memories, self-programming, daydreaming). Conversely, the network is inhibited when attention is externally focused (e.g., working memory, meditation; Brewer et al., 2013). It has been found that the PCC is not only related to emotional processing but also plays a key role in cognitive functioning and that abnormalities in the PCC are often associated with cognitive impairments, including memory function and concentration problems (e.g., Alzheimer’s disease, traumatic brain injury, ADHD; Leech and Sharp, 2014). At a physiological level, the posterior cingulate cortex shows abnormalities in metabolic levels and synaptic connections early in the brain of AD patients (Scheff et al., 2015). The slow-5 band CAP5 shows activation in the posterior cingulate cortex, angular gyrus and occipital gyrus and inhibition in the precentral gyrus and postcentral gyrus (Brodmann areas 3 and 4), which are associated with the primary motor and sensory cortex of the brain. However, the inhibitory brain regions in this band also contained several of the typical DMN-related brain regions described above. Furthermore, the correlation analysis revealed that all of the above CAPs showed significant correlations with subjects’ MMSE scores. Our findings are consistent with previous studies that have found differences in some dynamic indicators of the default mode network (DMN) and ventral/dorsal visual network (dorsal/ventral VN)-related CAP between AD and NC (Ma et al., 2020). The PCC, precuneus, angular gyrus and medial prefrontal lobes mentioned above belong to the typical DMN network, whereas the lingual gyrus, talar sulcus and cuneus belong to the typical dorsal and ventral visual networks. Damage to the DMN network and deterioration of higher visuospatial abilities have been recognized as early and prominent clinical signs of AD. Although the neurophysiological basis remains controversial, most previous visual task-based studies have concluded that this is due to dysfunction of the visual perceptual stream associated with cognition (Franceschi et al., 2007; Paxton et al., 2007). In addition, another fMRI study showed altered interactions between the PCC or precuneus and the visual perceptual network in MCI and AD compared to NC in a visual task (Krajcovicova et al., 2017). Our result is consistent with this finding and suggests that the interaction between the PCC or precuneus and the visual perceptual network is altered in AD and MCI patients. This has shown that the higher-order visual processing dysfunction seen in AD and MCI patients may arise from inefficient communication mechanisms between these regions.

Based on CAP analysis, delineating subbands was more helpful in distinguishing AD from NC. Our dynamic characterization of the CAP in the two groups of subjects in different frequency bands revealed that the only CAP that differed between AD and NC in the LFO band was CAP 3, while the number of CAP patterns with component differences increased to three in the slow-5 band and to five in the slow-4 band. These results suggest that the subband division may help to reveal differences in brain activity. Several previous studies (Han et al., 2011; Wee et al., 2012; Mascali et al., 2015; Yang et al., 2018) found that decomposing the BOLD signal into smaller bands and analyzing subbands would provide a more sensitive representation of the spatiotemporal information of brain activity, allowing for better identification of abnormalities in brain function in AD patients. Furthermore, our correlation analysis revealed that dynamic characterization of CAPs that showed significantly different between AD and NC groups almost realized significant correlations (positive or negative) with MMSE score. These studies suggest that CAP analysis has the potential to be a clinical indicator of AD.

## 5. Limitations and future directions

Various limitations need to be taken into consideration in future research. First, the current study investigated the subbands CAPs, the subbands definition might constrain the CAP results. More research is needed to investigate the subbands properties of CAPs by using wavelet-based or other frequency bands in the future. Second, further study should focus on using machine learning based on subbands CAP to distinguish the NC, AD, which result may be useful in the clinical diagnosis of AD. Finally, the relationship between subbands CAPs and physiological meaning is still unclear. More studies are needed to fully understand the physiological meaning of the CAPs. Hence, further studies that combine CAPs with genotype or bio-analyses are necessary to identify main reason behind the altered CAPs We also plan to apply frequency dependent whole-brain CAP method to other diseases, such as autism and major depressive disorder.

## 6. Conclusion

In this study, we used whole-brain CAP analysis in multiple frequency bands. The findings revealed that similar CAP spatial patterns are shown in different frequency bands, but the dynamic characterization of similar patterns in subbands was different. In addition, CAPs associated with the default mode network (DMN) and the ventral/dorsal visual network (dorsal/ventral VN) were altered significantly between the AD and NC groups. The findings also revealed that delineating subbands was more helpful in distinguishing AD from NC in terms of CAP.

## Data availability statement

The original contributions presented in the study are included in the article/supplementary material, further inquiries can be directed to the corresponding authors.

## Author contributions

S-PZ, YL, NY, and Z-GH designed the project. BM, S-PZ, CL, JJ, and TZ analyzed the data. S-PZ, YL, BM, C-WS, SA, NY, and Z-GH interpreted the results and wrote the manuscript. All authors participated in the revision of the manuscript, contributed to the article, and approved the submitted version. All authors provide approval for publication of the content and agree to be accountable for all aspects of the work in ensuring that questions related to the accuracy or integrity of any part of the work are appropriately investigated and resolved.

## Alzheimer’s Disease Neuroimaging Initiative

Data used in preparation of this article were obtained from the Alzheimer’s Disease Neuroimaging Initiative (ADNI) data base (https://adni.loni.usc.edu). As such, the investigators within the ADNI contributed to the design and implementation of ADNI and/or provided data but did not participate in analysis or writing of this report. A complete listing of ADNI investigators can be found at https://adni.loni.usc.edu/wpcontent/uploads/howtoapply/ADNI AcknowledgementList.pdf.

## Footnotes

^1^ https://adni.loni.usc.edu/
